# Minimally Invasive Arthroscopic Fixation of Scaphoid Nonunion in Adolescents Shows High Union Rates, Improved Function, and Low Morbidity

**DOI:** 10.1002/ars2.70008

**Published:** 2026-04-20

**Authors:** Ricardo Kaempf, Paula Fischer, William Brasil Souza, Victor Trivino, Pedro J Delgado, Sanjeev Kakar

**Affiliations:** ^1^ Santa Casa de Misericórdia Hospital of Porto Alegre Porto Alegre Brazil; ^2^ Madrid Montepríncipe University Hospital CEU San Pablo University Madrid Spain; ^3^ Department of Orthopaedic Surgery Mayo Clinic Rochester Minnesota U.S.A.

## Abstract

**Purpose:**

To evaluate the clinical, radiographic, and functional outcomes of arthroscopic treatment of scaphoid nonunion in adolescents, using cancellous bone grafting combined with headless compression screw fixation.

**Methods:**

This retrospective study included adolescent patients treated between 2015 and 2022 with arthroscopically assisted bone grafting and headless compression screw fixation for scaphoid nonunion. Inclusion criteria were nonunion ≥6 months after injury and minimum 12‐month follow‐up; exclusion criteria included degenerative wrist changes. Collected variables included demographic data, injury‐to‐surgery interval, radiographic parameters (including scapholunate angle), time to union, pain scores (visual analog scale), functional outcomes (QuickDASH), and range of motion. Statistical analysis showed significant improvements in visual analog scale and QuickDASH scores (*P* < .05).

**Results:**

Fifteen patients (14 males; mean age 16 years, range 13‐19) met inclusion criteria. The mean follow‐up was 29.2 months. All patients achieved bone union, with an average healing time of 5.7 weeks. Pain (visual analog scale) improved from 2.6 to 0.8, and QuickDASH scores improved from 26.5 to 7.9. The scapholunate angle improved from 60.6° to 46.6°, with stable range of motion postoperatively. No major complications or reoperations occurred. Minimal clinically important difference analysis confirmed meaningful clinical improvement in 93% of patients for pain and 87% for QuickDASH.

**Conclusions:**

Arthroscopic treatment of scaphoid nonunion in adolescents shows high union rates, improved function, and low morbidity.

**Level of Evidence:**

Level IV, therapeutic retrospective case series.

## Graphical abstract

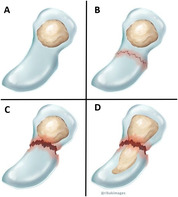

The pediatric scaphoid exhibits distinct developmental and vascular characteristics that influence injury patterns and outcomes. The ossification center of the scaphoid appears at around 5 years of age, with calcification progressing from distal to proximal. This explains why, in early childhood, the scaphoid is more susceptible to chondral injuries than to fracture.^1^ Eighty percent of fractures occur in the distal third, a region that is typically well vascularized, thereby increasing their propensity for union.^1^, ^2^, ^3^, ^4^ Given this, the rate of scaphoid nonunion (SN) is rare, with an incidence of 0.8% to 1.5% following conservative treatment.^5^ Risk factors include male sex, adolescence, high‐energy trauma, proximal fractures, and delayed treatment initiation.^3^, ^5^, ^6^, ^7^

In adolescents, due to greater involvement in contact sports and high‐energy trauma, the pattern of scaphoid injuries now resembles that seen in adults, with more waist and proximal pole fractures. In adults, untreated SN leads to instability and degenerative scaphoid nonunion advanced collapse wrist changes.^8^ Therefore, the goal of surgery is to achieve union and restore carpal alignment. In adolescents, however, the natural history of SN remains unclear.^3^, ^5^, ^6^

Early surgical intervention is recommended for unstable or symptomatic cases, and multiple techniques (open and arthroscopic) have been described with favorable results.^7^, ^9^, ^10^, ^11^, ^12^ Recent studies emphasize the advantages of arthroscopy, including less invasiveness, lower morbidity, and preservation of vascularity and proprioception.^4^, ^9^, ^13^, ^14^, ^15^, ^16^, ^17^

The purpose of this study was to evaluate the clinical, radiographic, and functional outcomes of arthroscopic treatment of SN in adolescents using cancellous bone grafting combined with headless compression screw (HCS) fixation. The hypothesis of this study was that arthroscopic bone grafting and screw fixation provide high union rates, improved pain and function, and restoration of carpal alignment, with low morbidity and complications, in adolescent patients with SN.

## METHODS

This retrospective study included patients treated between 2015 and 2022 for SN with arthroscopic bone grafting and headless screw fixation. The study was approved by the institutional ethics committee.

**FIGURE 1:**
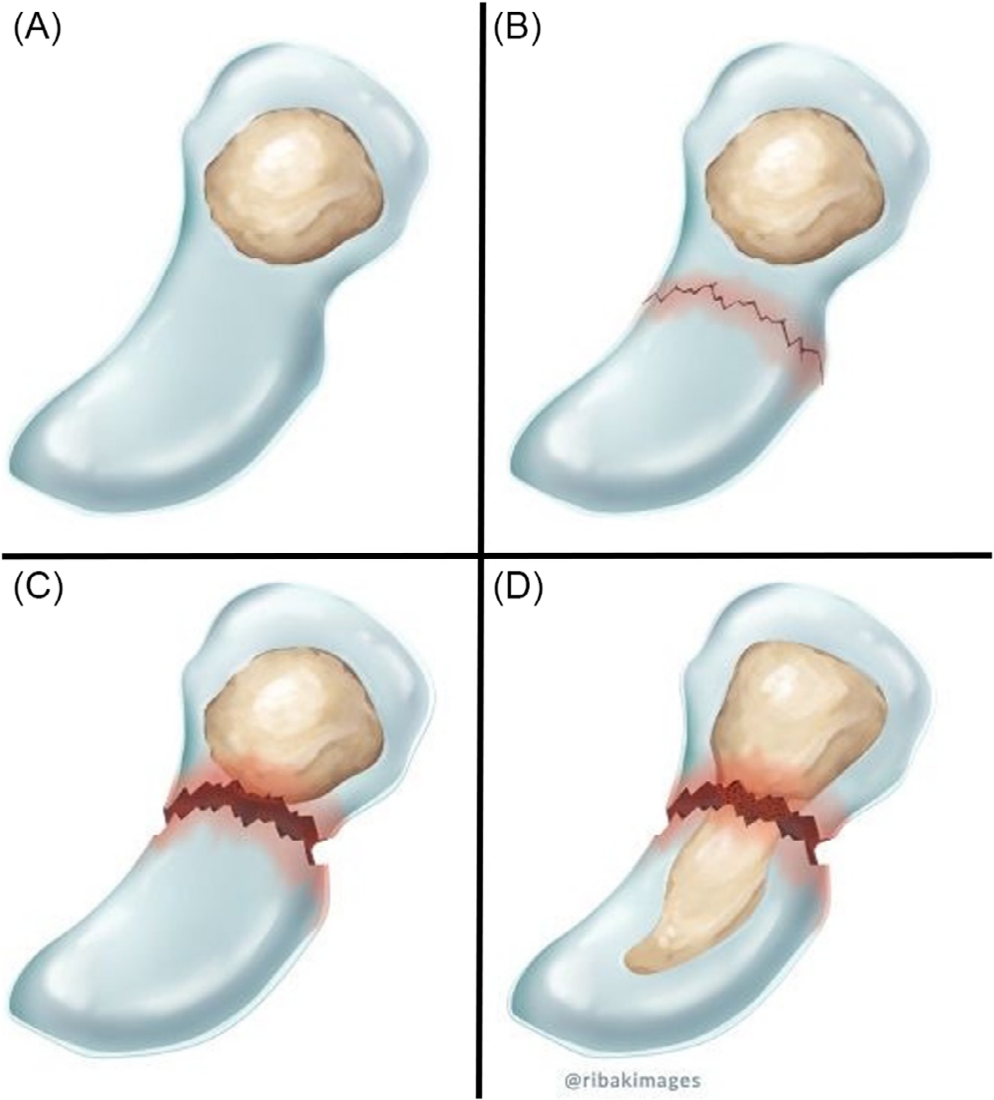
D’Arienzo classification for scaphoid fractures in children. Normal scaphoid (A); pure chondral fracture—type I (B); osteochondral fracture—type II (C); fracture through the osseous portion—type III (D).

Inclusion criteria: SN ≥6 months postinjury; minimum follow‐up of 12 months. Exclusion criteria consisted of degenerative wrist changes and prior scaphoid surgery. We used the D’Arienzo classification for pediatric scaphoid fractures, which divides them into 3 types: type 1 is a pure chondral fracture, type 2 is an osteochondral fracture, and type 3 is a bony lesion (Figure 1).^18^, ^19^

The Herbert and Fisher classification was used to categorize the type of nonunion: D1 indicates a fibrous lesion, D2 an unstable nonunion, D3 a nonunion with sclerotic edges, and D4 with avascular necrosis.^20^, ^21^

The degree of instability was measured using the scapholunate angle via the picture archiving and communication system (Terch Heim, Seoul, South Korea).^3^

Before surgery, a functional assessment was conducted using the QuickDASH (Disabilities of the Arm, Shoulder, and Hand) score and the visual analog scale for pain (0 representing no pain and 10 representing the worst possible pain). These were recorded preoperatively and at final follow‐up.^9^

Patients were also assessed for range of motion (ROM)—flexion, extension, radial deviation, and ulnar deviation—measured in degrees using a standard goniometer and rounded to the nearest multiple of 5°.

Evaluations were carried out preoperatively and at final follow‐up, performed by one of the authors. Clinical outcomes, time to bone union, time to return to sports, and any surgical complications were analyzed. Computed tomography (CT) scans were performed preoperatively and at final follow‐up to confirm union.

Postoperatively, radiologic studies of the wrist with scaphoid‐specific views were performed every 2 weeks until consolidation. Bone union was defined clinically by the absence of pain upon palpation in the anatomical snuffbox, and radiographically by the presence of a bony bridge with more than 50% transverse trabeculation between the main bone fragments in all radiographic views or at least in 2 CT slices.^22^, ^23^

The procedure is performed using a technique similar to that described for adult patients.^10^ The patient is placed in the supine position under brachial plexus block anesthesia with sedation and a pneumatic tourniquet. Finger traps are applied to the second and fourth fingers for continuous traction using a wrist arthroscopy tower. Standard radiocarpal (RC) and midcarpal (MC) arthroscopic portals are created for complete joint evaluation.

The first step is to inspect the RC and MC joints to assess for associated lesions, such as chondral injuries, intrinsic ligament damage, and triangular fibrocartilage complex tears.

**FIGURE 2:**
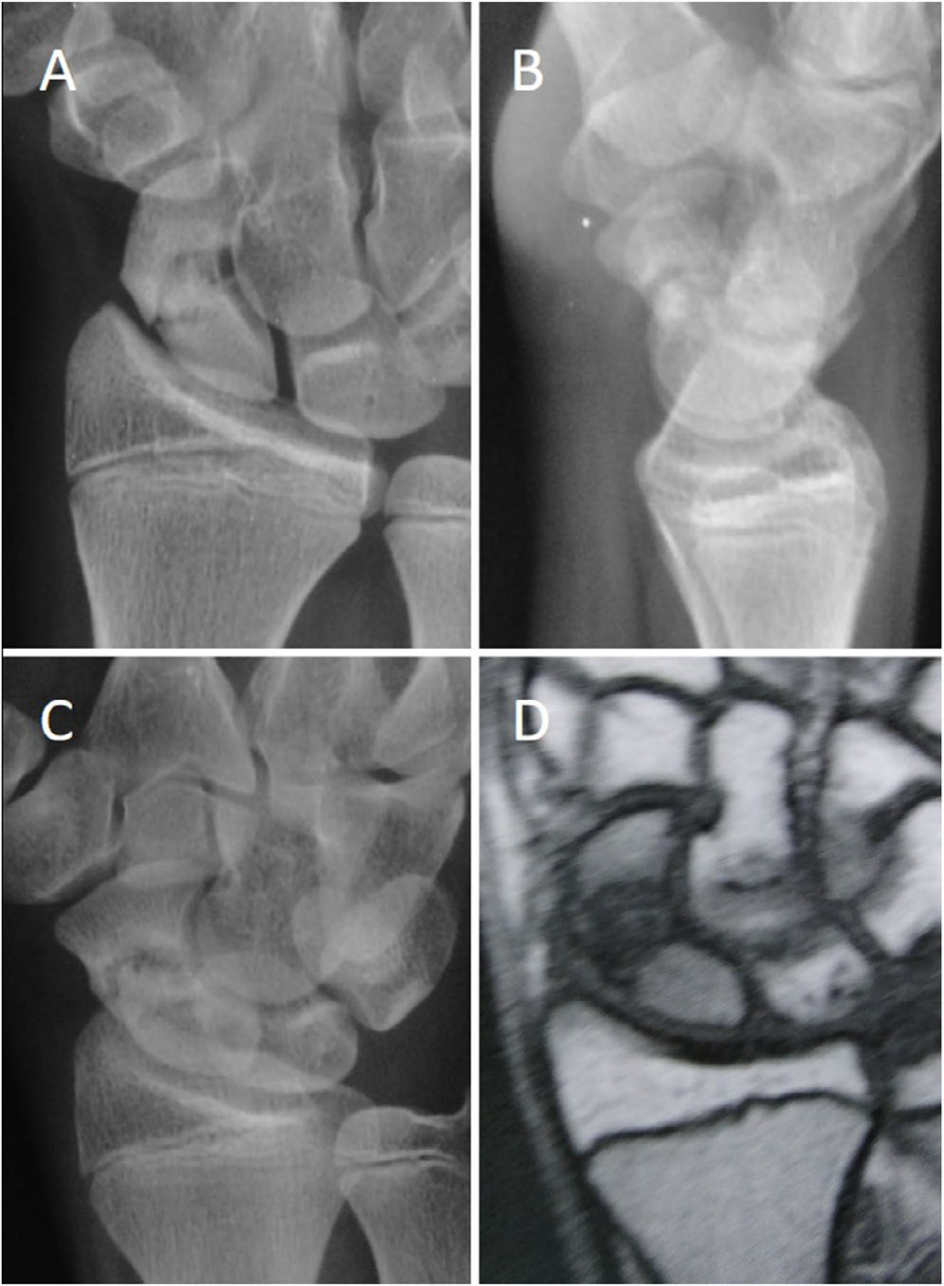
Fourteen‐year‐old male patient with a scaphoid fracture sustained 6 months prior, which progressed to nonunion. Anteroposterior (A), lateral (B), and oblique (C) radiographs confirm the lesion. Magnetic resonance imaging (D) shows no vascular compromise of the bone fragments.

**FIGURE 3:**
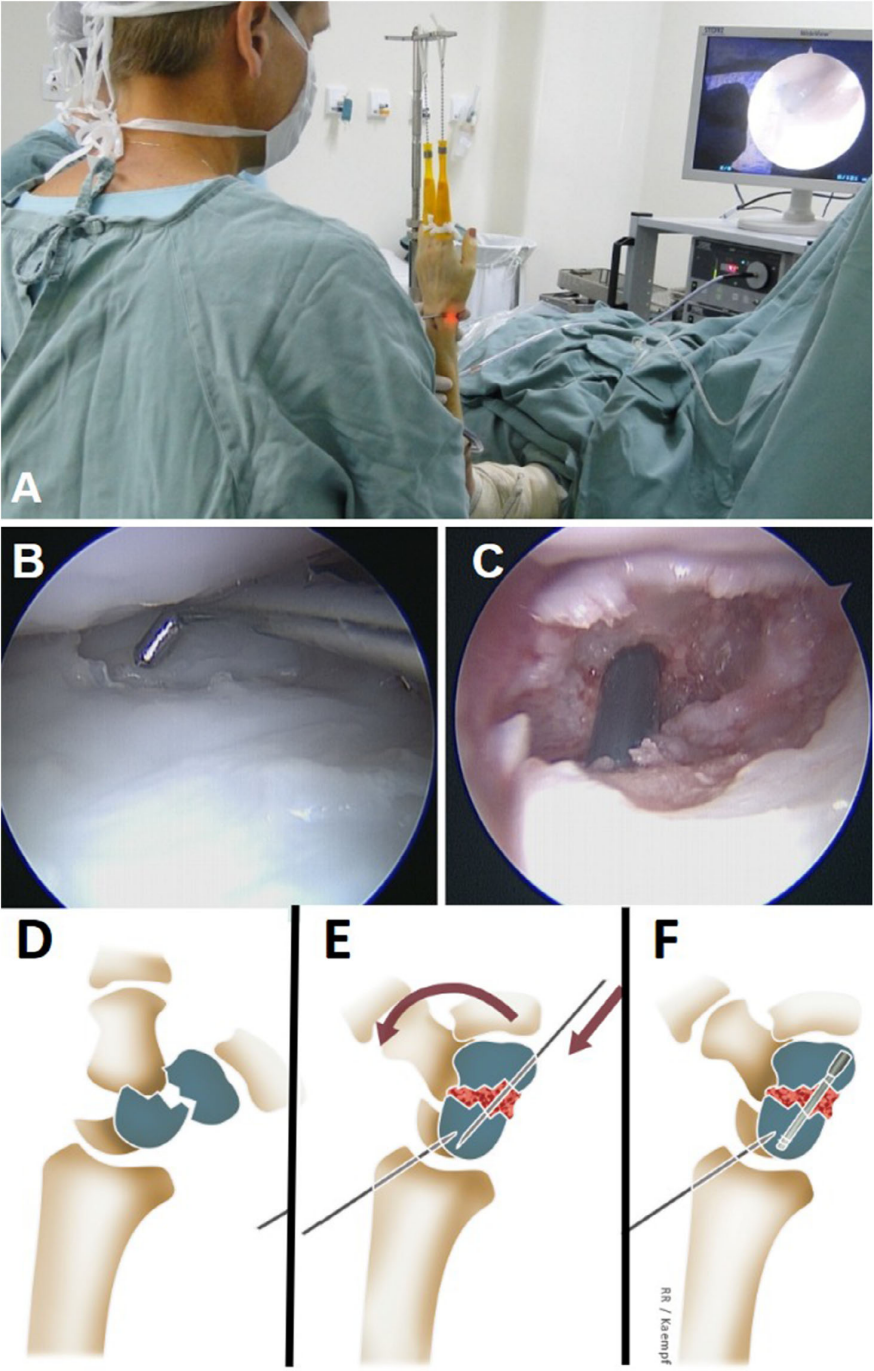
Arthroscopic surgical treatment. Patient positioned with the upper limb stabilized by a traction tower (A). Observation of the nonunion site through the midcarpal portals (B). Visualization of the headless compression cannulated screw through the previously debrided nonunion focus (C). Correction of dorsal intercalated segment instability of the proximal carpal row using the Linscheid maneuver. Wrist flexion reduces the lunate and proximal pole of the scaphoid (D); wrist extension reduces the flexion deformity of the scaphoid (E); percutaneous fixation of the lesion with a headless compression cannulated screw (F).

To access the nonunion site, the arthroscope is placed in the ulnar MC portal, and instruments are used through the radial MC portal. A probe, arthroscopic shaver, curettes, and curved osteotomes are used. The nonunion site is first debrided with a periosteal elevator and shaver to return to healthy cancellous bone. A dry arthroscopy technique is used, with saline solution only for joint cleaning and tissue fragment removal. In adolescents, due to the thick cartilage layer, locating the nonunion site can be challenging (Figures 2 and 3). In such cases, we recommend placing a needle through the RC 1‐2 portal, directed from radial to ulnar and dorsal to volar, to identify the lesion site using fluoroscopic guidance.

After cleaning the nonunion site, in unstable cases with humpback deformity, the next step is correcting dorsal intercalated segment instability of the proximal carpal row. This is achieved with the Linscheid maneuver,^24^ where wrist flexion aligns the lunate with the proximal pole of the scaphoid. A 1.5‐mm Kirschner wire (K‐wire) is then percutaneously inserted dorsoradially from the distal radius into the lunate. This maintains the lunate in neutral rotation.

**FIGURE 4:**
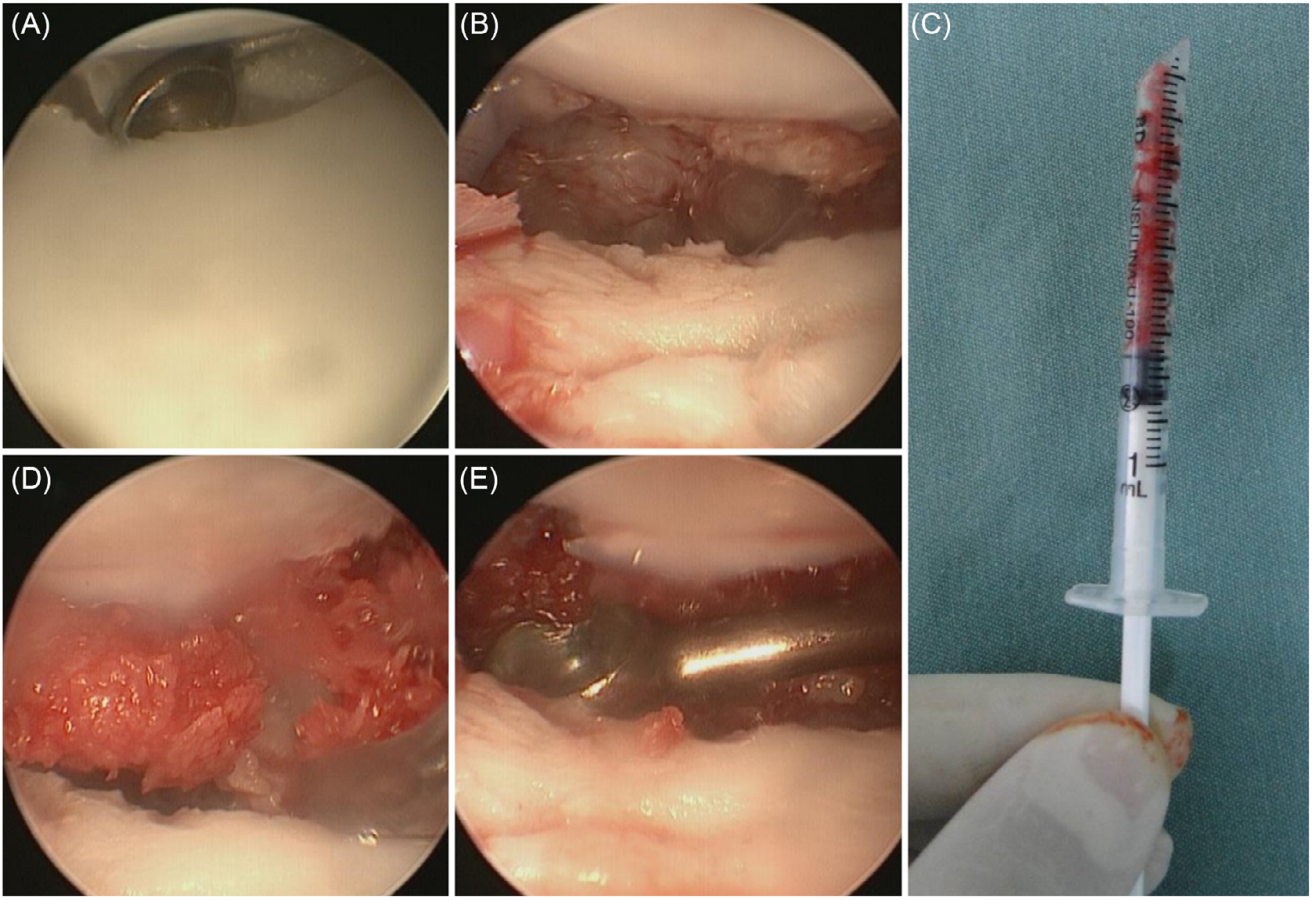
Steps of the arthroscopic procedure for treating scaphoid nonunion in adolescents. Debridement of the lesion with a bone curette (A); correction of the flexion deformity and scaphoid lengthening using the Linscheid maneuver (B); placement and impaction of the cancellous bone graft with the aid of a syringe (C‐E).

Next, autologous cancellous bone graft is harvested from the distal radius in stable cases or from the iliac crest in cases with bone loss or deformity and is packed into the defect. We use a 1‐mL syringe with the tip cut at an angle to facilitate insertion and positioning of the graft. For middle‐third nonunions, we prefer debriding the radial portion of the nonunion and inserting the graft through the RC 1‐2 or 2 R portal, which provides direct access to the radial‐volar aspect of the scaphoid—typically the area with the greatest bone loss.^22^ Alternatively, the radial MC portal can be enlarged to accommodate the syringe. Once the graft is in place, it is impacted using the back of a curette (Figure 4).

**FIGURE 5:**
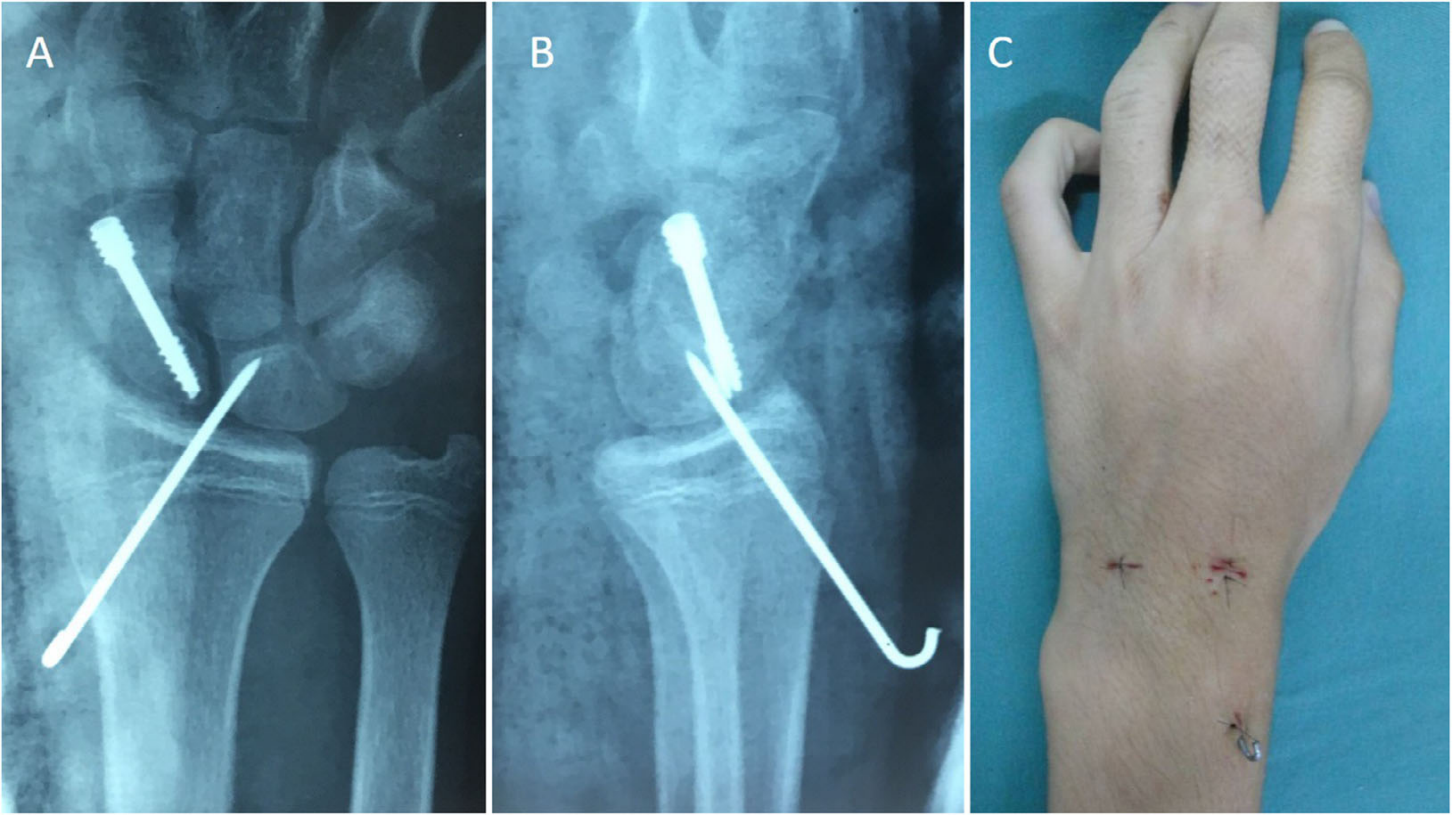
Follow‐up radiographs at 2 weeks showing proper alignment and fixation of the scaphoid (A,B). Dorsal view of the wrist showing the midcarpal radial and ulnar arthroscopy portals and the Kirschner wire used in the Linscheid maneuver (C).

**FIGURE 6:**
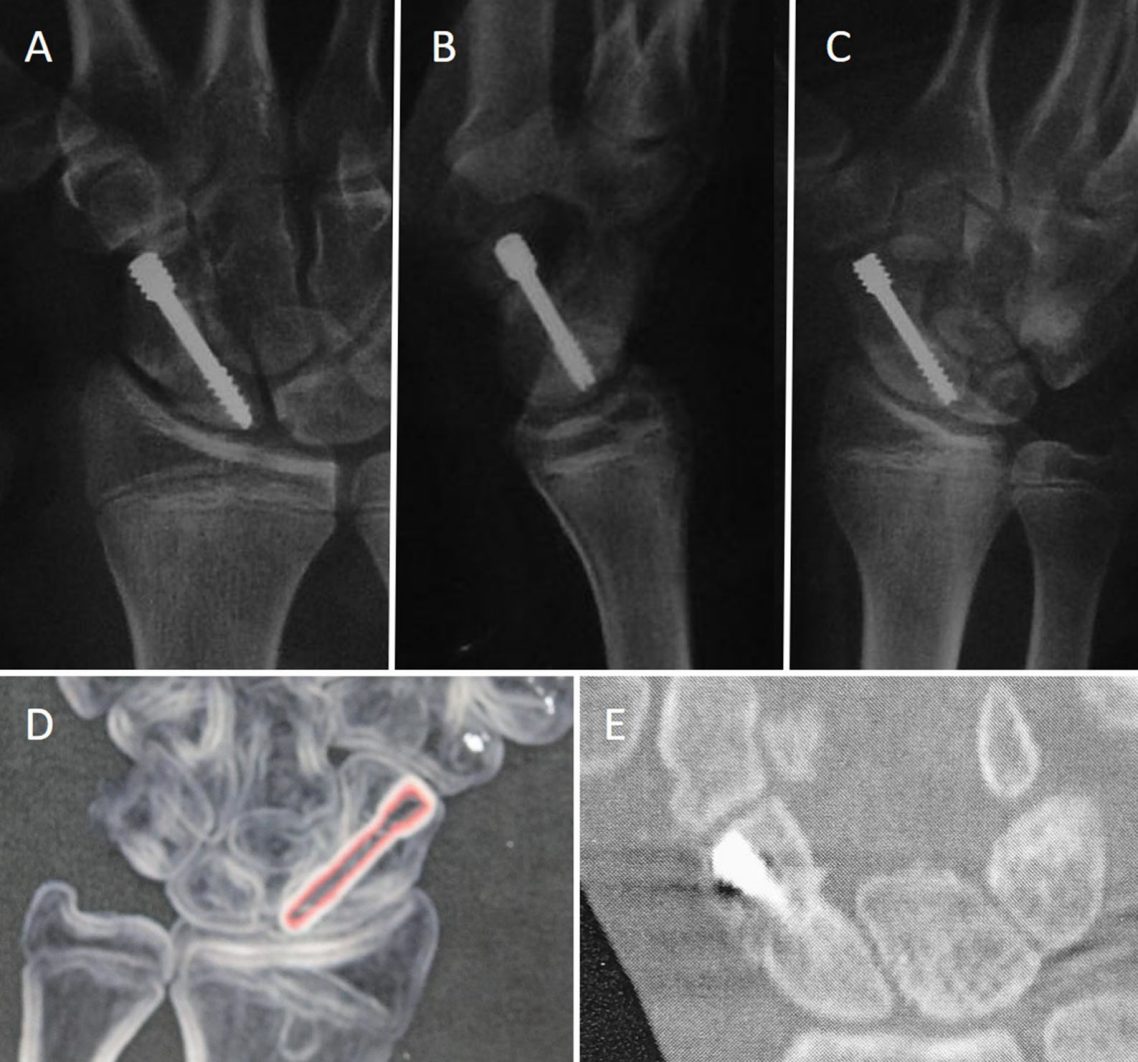
Follow‐up radiographs in anteroposterior (A), lateral (B), and oblique (C) views showing bone healing 4 weeks after surgery. Computed tomography scan confirms consolidation (D,E).

The final step is reducing the distal scaphoid pole and fixing it with an HCS 2.4 to 3.0 mm. In middle and distal‐third nonunions, we use a percutaneous retrograde fixation technique. The wrist is taken out of the traction tower and extended, ulnarly deviated and thumb hypersupinated to ensure reduction of the scaphoid. A guide K‐wire is inserted just radial to the trapezium. Once fluoroscopy confirms the wire location within the central longitudinal aspect of the scaphoid, it serves as a guide for screw placement. A second derotational, parallel K‐wire is inserted in the same manner as the first, within the scaphoid (Figures 5 and 6).

**FIGURE 7:**
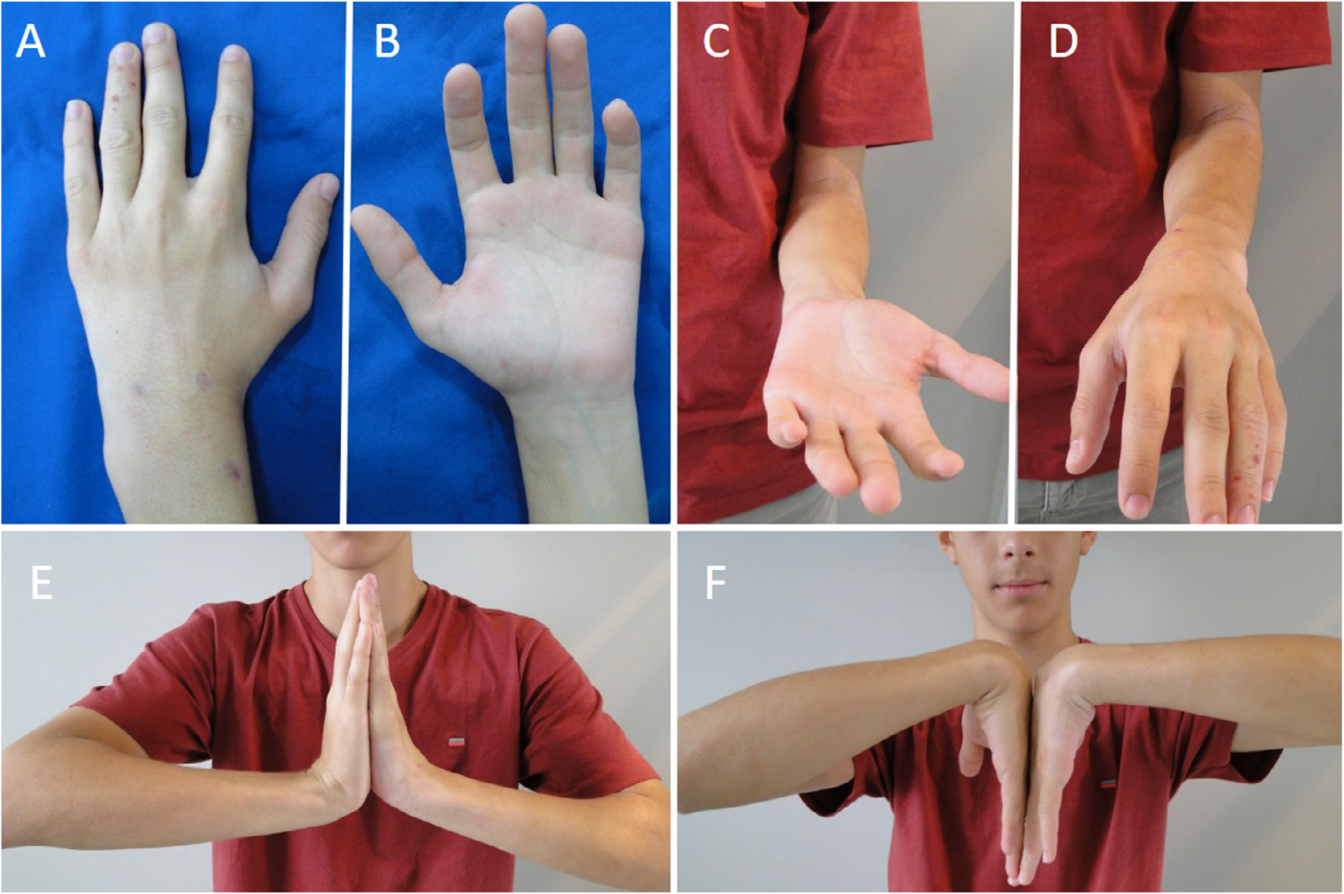
Six months after surgery, the patient shows arthroscopy portal scars on the dorsal wrist and the entry point of the Kirschner wire (A), as well as the entry site of the screw on the volar side (B). Complete recovery of wrist mobility is also observed (C‐F).

This procedure is typically performed on an outpatient basis. After surgery, the wrist is immobilized in a short arm plaster splint, leaving the fingers and thumb free, for 2 weeks. The K‐wire fixing the radius to the lunate is left in place, buried, for 4 weeks. After this period, the splint is removed, and the patient is referred to therapy. During rehabilitation, patients are instructed to wear a removable wrist orthosis during activities requiring force until bone consolidation is confirmed (Figure 7).

### Statistical Analysis

Statistical analysis was performed using paired t‐tests to compare pre‐ and postoperative visual analog scale, QuickDASH, and ROM measures. A *P* value < .05 was considered statistically significant. Descriptive statistics were reported as means and ranges.

## RESULTS

Using the above‐mentioned criteria, 32 patients were identified as having undergone the described treatment, and 15 were available for final follow‐up. There were 14 male patients, and mean age was 16.0 years (range: 13‐19 years). The average time between fracture and surgery was 10.0 months (range 6‐18 months). According to the D’Arienzo classification, all initial fractures were type 3 (bony lesions). Regarding the type of SN, using the Herbert and Fischer classification, 11 were type D2 and 4 were D3.^21^

As for associated injuries found during the procedure, no chondral lesions, scapholunate ligament injuries, or triangular fibrocartilage complex injuries requiring repair were observed.

The average follow‐up was 29.2 months, ranging from 12 to 60 months. Bone healing was achieved in all patients (100%) with an average time of 5.7 weeks, ranging from 4 to 8 weeks.

At final evaluation, improvements were observed in pain symptoms, radiological parameters, and functional criteria according to the QuickDASH questionnaire, while the ROM remained stable compared with preoperative measurements (see Tables 1 and 2).

**TABLE 1:** Preoperative Evaluation

Patient	Gender	Age	Side	Time Since Injury, m	D’Arienzo Classification	Herbert Classification	Scapholunate Angle, Pre	Pain, Pre	Flexion, Pre	Extension, Pre	Radial Deviation, Pre	Ulnar Deviation, Pre	DASH, Pre
1	M	18	L	15	3	D2	60	3	85	75	20	30	28
2	M	17	R	12	3	D3	65	3	75	65	25	25	32
3	M	15	R	9	3	D2	60	4	80	65	20	30	46
4	M	15	R	7	3	D2	80	2	70	55	20	20	22
5	M	14	L	6	3	D2	55	1	90	80	25	30	18
6	M	13	L	8	3	D2	70	3	75	65	20	35	50
7	M	19	L	18	3	D3	60	3	80	70	25	30	33
8	M	14	L	6	3	D2	60	2	70	70	20	30	36
9	M	18	R	12	3	D3	45	4	70	65	25	25	16
10	M	17	R	10	3	D2	65	2	90	75	25	35	22
11	M	16	R	6	3	D2	55	1	80	75	20	25	19
12	F	18	L	12	3	D2	50	2	90	80	25	35	15
13	M	14	R	8	3	D2	70	4	80	70	20	25	28
14	M	15	R	10	3	D2	60	3	80	65	20	25	21
15	M	17	R	12	3	D3	55	2	90	80	30	30	12

DASH, Disabilities of Arm, Shoulder and Hand; F, Female; L, left, m, month; M, male; R, right.

**TABLE 2:** Postoperative Outcomes

Patient	Type of Fixation	Graft Bone	Follow‐Up, m	Scapholunate Angle, Post	Pain, Post	Flexion, Post	Extension, Post	Radial Deviation, Post	Ulnar Deviation, Post	Bone Healing wk	Return to Sports, wk	DASH, Post
1	Herbert	Iliac	48	50	1	80	75	30	30	6	6	8
2	Herbert	Iliac	24	45	0	80	75	25	30	4	4	14
3	HCS	DR	12	50	1	75	70	25	25	6	8	9
4	Herbert	Iliac	18	50	2	75	65	20	30	8	10	7
5	Herbert	Iliac	36	45	0	90	80	25	25	4	8	4
6	HCS	Iliac	24	55	0	85	70	25	35	6	6	13
7	Herbert	Iliac	36	45	1	80	65	25	30	5	6	9
8	ACUTRAK	Iliac	60	50	1	75	70	30	30	5	12	12
9	Herbert	Iliac	12	45	2	80	75	25	25	6	8	5
10	Herbert	Iliac	18	50	1	75	75	30	35	7	4	9
11	HCS	DR	48	45	0	70	70	20	30	5	12	3
12	Herbert	Iliac	36	40	0	85	80	30	30	4	6	4
13	Herbert	DR	18	45	2	85	75	20	25	6	9	10
14	Herbert	Iliac	12	40	1	85	70	25	35	8	5	5
15	HCS	DR	36	45	0	90	80	25	30	6	6	7

DASH, Disabilities of Arm, Shoulder and Hand; DR, radial deviation; HCS, headless compression screw; m, month; wk, week.

Pain measured on the visual analog scale improved from a mean of 2.6 (range: 1‐4) to 0.8 (range: 0‐2). Functional scores on the QuickDASH improved from an average of 26.5 (range: 12‐50) to 7.9 (range: 4‐12).

Regarding carpal instability measured by the scapholunate angle, there was improvement from a preoperative mean of 60.6° (range: 45°‐80°) to 46.6° (range: 40°‐55°).

Average flexion ROM was 80.3° (range: 70°‐90°) preoperatively to 80.6° (range: 70°‐90°) postoperatively. Extension improved from 70.3° (range: 65°‐80°) to 73.0° (range: 65°‐80°), respectively. Ulnar deviation improved from 28.6° (range: 25°‐35°) to 29.6° (range: 25°‐35°). Radial deviation improved from 22.6° (range: 20°‐30°) to 25.3° (range: 20°‐30°).

One patient developed a skin burn from overheating of the shaver motor, which healed with routine wound care. None of the cases were converted to an open procedure or required secondary surgery.

## DISCUSSION

The main finding of this study is that arthroscopic bone grafting and HCS fixation for SN in adolescents resulted in 100% union, significant functional improvement, and minimal complications. These outcomes are consistent with prior adult studies showing high union rates and favorable recovery after arthroscopic management.^7^, ^9^, ^15^

Determining the best treatment for SN in children and adolescents remains challenging. This is due to the difficulty in conducting comparative studies—given the small number of cases—and the favorable outcomes observed with a variety of methods, which range from conservative treatment to percutaneous fixation with K‐wires or screws to open procedures using free or vascularized bone grafts.^1^, ^25^ In this study, we report on the role of arthroscopic bone grafting and fixation with good outcomes.

Minimal clinically important difference (MCID) analysis was included to provide a patient‐centered interpretation of clinical improvement. Ninety‐three percent of patients exceeded the MCID threshold for pain, and 87% for QuickDASH scores, indicating meaningful clinical benefit beyond statistical significance.

The use of wrist arthroscopy in treating traumatic and degenerative carpal pathologies is well established.^17^, ^26^ Its benefits include lower morbidity, quicker bone healing, restoration of normal carpal anatomy, and the ability to treat associated injuries during the same procedure.^4^, ^6^, ^9^, ^22^, ^27^ It also preserves the joint capsule and carpal ligaments, structures that, when damaged in open procedures, can impair proprioception and further compromise scaphoid vascularity.^8^ The arthroscopic surgical technique presented here for adolescent patients with SN is similar to that described in adults, where many studies have reported favorable outcomes.^9^, ^16^, ^17^ In this study, the average time to union was 5.7 weeks.^6^, ^21^, ^22^, ^23^

Open techniques using corticocancellous bone grafts require more extensive exposure and carry risks such as fibrosis, stiffness, infection, loss of proprioception, graft protrusion, and greater disruption of bone vascularity—factors that extend healing time (reported between 12 and 19 weeks).^1^, ^6^, ^8^ Our results show that arthroscopy can also restore carpal alignment, as seen in the reduction of the scapholunate angle from 60.6° preoperative to 46.6° postoperative.

There are few published studies on the use of arthroscopy in children and adolescents. Lee and Kim described its use with bone grafts and K‐wire fixation in 3 patients, achieving union in an average of 10.3 weeks. They also noted partial scapholunate ligament injuries in all cases—none of which were observed in our series.^9^

There is no consensus on the ideal type of bone graft for unstable SN. The use of autologous nonstructural cancellous bone grafts has proven effective in adults.^9^, ^10^, ^22^ Cohen et al. reported excellent clinical evolution and 100% healing in 2 years in patients with collapsed SN using only autologous nonstructural grafts.^1^ Park et al. treated 61 patients (52% stable and 48% unstable) using autologous cancellous grafts and K‐wire fixation, achieving 88.2% union in stable cases and 83.9% in unstable ones, with no significant differences between groups.^23^ Kim et al. reported 86% healing in 36 patients treated arthroscopically with autologous cancellous bone grafts, with an average healing time of 11 weeks.^23^ Wong and Ho used allogeneic nonstructural bone grafts combined with arthroscopic fixation, achieving 91.2% union in 12 weeks, with no method‐related complications.^20^ However, little has been published about such graft use in younger patients.

In our series, we preferred using autologous iliac crest cancellous bone grafts in cases with deformity and bone resorption due to their better quality and quantity. It also avoids altering the distal radius metaphysis, preserving it for potential future vascularized grafts. In stable SN with minimal deformity, cancellous grafts from the distal radius metaphysis may be a good alternative, especially in older adolescents with closed growth plates, also providing vascular stimulation through metaphyseal decompression.

Internal fixation methods of scaphoid fractures and nonunions have evolved, including K‐wires, plates, staples, and screws. HCS are widely used for providing compression and stability. Oestreich et al. noted theoretical concerns about damage to the ossification center cartilage from such screws but found them safe and effective in children as young as 9, without any complications.^7^

In arthroscopy, potential complications are mainly related to portal creation and percutaneous fixation.^4^ Basso et al., in a systematic review of 18 articles, found no major complications but noted minor technical issues in 2 studies, including radial nerve sensory neuropraxia (4 cases) and superficial infections (3 cases). In our series, there were no secondary surgeries. One patient had a superficial skin burn from the shaver, which healed with dressings. This complication can be avoided by regularly irrigating with saline to cool tissues and equipment.^28^

We acknowledge that this technique may be demanding and, especially in pediatric patients, requires prior wrist arthroscopy training. Surgeons should be experienced with open techniques and be ready to convert if needed.

### Limitations

The limitations of this study include its retrospective design, small sample size, and absence of a control group for direct comparison with open techniques. Additionally, the results may not be generalizable to adults due to differences in bone biology. Although we attempted to disaggregate data by sex, the small number of female patients (n = 1) prevented meaningful comparative analysis. Another limitation is the lack of long‐term follow‐up to assess potential late degenerative changes.

## CONCLUSIONS

Arthroscopic treatment of SN in adolescents shows high union rates, improved function, and low morbidity.

## DISCLOSURES

The authors (R.K., P.F., W.B.S., V.T., P.J.D., S.K.) declare that they have no known competing financial interests or personal relationships that could have appeared to influence the work reported in this paper.
